# SmartHerd management: A microservices‐based fog computing–assisted IoT platform towards data‐driven smart dairy farming

**DOI:** 10.1002/spe.2704

**Published:** 2019-05-16

**Authors:** Mohit Taneja, Nikita Jalodia, John Byabazaire, Alan Davy, Cristian Olariu

**Affiliations:** ^1^ Emerging Networks Laboratory, Telecommunications Software and Systems Group, Department of Computing and Mathematics, School of Science and Computing Waterford Institute of Technology Waterford Ireland; ^2^ CONNECT ‐ Centre for Future Networks and Communications Dublin Ireland; ^3^ Innovation Exchange IBM Ireland Dublin Ireland

**Keywords:** cloud computing, dairy farming, data analytics, data‐driven, fog computing, Internet of Things (IoT), microservices, smart farm

## Abstract

Internet of Things (IoT), fog computing, cloud computing, and data‐driven techniques together offer a great opportunity for verticals such as dairy industry to increase productivity by getting actionable insights to improve farming practices, thereby increasing efficiency and yield. In this paper, we present SmartHerd, a fog computing–assisted end‐to‐end IoT platform for animal behavior analysis and health monitoring in a dairy farming scenario. The platform follows a microservices‐oriented design to assist the distributed computing paradigm and addresses the major issue of constrained Internet connectivity in remote farm locations. We present the implementation of the designed software system in a 6‐month mature real‐world deployment, wherein the data from wearables on cows is sent to a fog‐based platform for data classification and analysis, which includes decision‐making capabilities and provides actionable insights to farmer towards the welfare of animals. With fog‐based computational assistance in the SmartHerd setup, we see an 84% reduction in amount of data transferred to the cloud as compared with the conventional cloud‐based approach.

## 1 INTRODUCTION

The demand for dairy products is rapidly rising due to an ever increasing population coupled with an increase in income per capita.^1^ The consumption of dairy and dairy products is more in developed countries as compared with developing countries, but this gap is narrowing with rising incomes, population growth, urbanization, and changes in diet.^2^ Over 80% of the dairy production in developing countries is accounted to “smallholder farming” or “family farming,” mostly reliant on traditional practices with insignificant technology penetration. Catching up with the increasing production requirements requires better technological platforms that optimize production, even in remote small‐scale infrastructures with resource and development constraints. The increase in demand due to factors such as an evolving middle class^3^ presents an opportunity of specific interest to pasture‐based dairy farming industries like those present in Ireland, New Zealand, and Australia, as it is essential to increase milk productivity and yield to have a substantial presence in the market. This becomes even more important for fast growing and developing economies such as India, where a large percentage of population is vegetarian, and consumers look to dairy and its products to sustain their dietary requirements, especially for protein and calcium–based needs.

Dairy farms have all the constraints of a modern business, ie, a fixed production capacity, a herd to be managed, and farm labor, which is often quite expensive. They face several challenges to maintain their business, including labor shortages, climate changes, land availability, herd management, etc. With such constraints, dairy farmers majorly look to have two controls to increase productivity and profit of their dairy farm business:

- Monitoring the welfare of their livestock for anomalies and diseases, so as to detect health issue early and prevent loss.
- Improve cattle reproduction and milk yield by accurate heat detection (oestrus) so as to increase the size of the farm for further profitability.

It is expected that opting for smart dairy farming principles, which unifies Internet of Things (IoT), data analytics, fog computing, and cloud computing, will assist dairy farmers to overcome these challenges and help move the dairy industry towards sustainable growth in both developing and developed countries.^4^ Automating various processes on the farm such as heat detection (oestrus) for accurate reproduction, health assessment (lameness, etc), and monitoring the well‐being of animals, is an answer to labor shortage and is realized via data obtained from wearable devices worn by the livestock.^5^

While there have been several approaches towards smart farming by an active incorporation of information and communication technology (ICT) in the agricultural industry, a vast majority of them still remain practically unimplemented by virtue of the fact that a farm environment is much unlike an ideally “connected” IoT use case. The true penetration of ICT in the most granular levels of global farming diaspora depends on the transformative adaptability of the IoT ecosystem, wherein a shift is required from a predominantly urban context towards a more flexible solution that addresses the unique environmental, temporal, and spatial challenges of remote farm locations.

Our work is motivated by the need to enable data‐driven dairy farming by extracting timely value out of the data by designing suitable models and analytics, and providing different controls to the farmer and other stakeholders to increase productivity and yield, thus helping farmers adopt the best farming practices for the overall benefit. It is crucial to devise a flexible data‐driven solution that enables pervasive data collection and processing in remote farm infrastructures, with a system better adapted to performing critical operations even during outages and bouts of intermittent Internet connectivity.

In this work, we address these issues and propose the emerging fog computing infrastructure as a key enabler for IoT applications and services in situational scenarios of constrained Internet connectivity. We envision a microservices‐based approach to assist applications in such a hybrid fog‐cloud environment, and propose SmartHerd, which is an IoT platform solution that addresses the connectivity and animal welfare in a smart dairy farming scenario.

The paper has been further structured as follows. Section 2 presents the literature review, background, and motivation. Section 3 outlines limits, opportunities, and challenges in the penetration of ICT in agriculture. Section 4 presents the service orientation in the IoT ecosystem and microservices‐based architecture. Section 5 presents the proposed SmartHerd management IoT platform. Section 6 presents the results and analysis. Section 7 outlines the learnings and practical experiences from the real‐world deployment, and Section 8 presents the conclusion.

## 2 LITERATURE REVIEW, BACKGROUND, AND MOTIVATION

There have been proposed systems in industry^6^, ^7^, ^8^ as well as in academia^9^, ^10^, ^11^, ^12^ for animal health management in dairy farms. A study by Steeneveld and Hogeveen^13^ and that by Andonovic et al^14^ give an overview of the sensor systems available for health monitoring of animals in dairy farms.

Along with the proposed systems and solutions, there has also been research in integrating technologies to increase productivity and sustainable growth in agriculture, ranging from improving wireless network connectivity in economically underprivileged areas^15^, ^16^, ^17^ to data mining and analytics for agricultural applications^18^, ^19^, ^20^ to providing decision control in variable environments under constraints.^21^, ^22^, ^23^ A review by Wolfert et al^24^ shows that predictive insights in farming operations drive real‐time operational decisions, and redesigns business processes for the benefit of various stakeholders in a farming landscape, and that the influence of such systems goes beyond primary production, to the entire supply chain. However, the survey by Rutten et al^25^ identifies a serious lack of analytics and intelligence in these systems, thus leading to gaps between the desired requirement of the system and proposed solutions. It articulates the need and requirement of intelligence to be present on the premises, in the on‐farm systems.

As a consequence, attention is being drawn towards designing systems with intelligence and data analytics capability being present on premises,^26^ and utilizing fog computing comes to shore with those objectives in mind. As per the recent architecture released by OpenFog Consortium,^27^ fog should be seen as a horizontal architecture that distributes computation, communication, control, and storage closer to end users along the cloud‐to‐things continuum, and supports a variety of vertical domains of applications. Fog computing is the approach of providing intelligence and processing capabilities closer to the network edge.^28^, ^29^ While it was initially aimed to cater to latency sensitive and time‐critical applications and use‐case scenarios, we believe that fog computing will act as an active enabler for computing systems, applications, and services that suffer from constrained and unreliable cloud connectivity.

In our case, considering that the farm locations are usually remote with intermittent Internet connectivity, dependence on the cloud for pervasive monitoring and computation is less reliant due to a variety of connectivity issues and other associated factors such as high response latency and high bandwidth requirements.^30^ In such situations, fog computing can ensure high availability and less reliance on remote cloud infrastructures. Such a system becomes even of more importance with adverse weather conditions such as during storms and hurricanes,^31^, ^32^ when the Internet services get interrupted and affected for various reasons. The efficient use of available computational resources realized by means of fog computing, in turn, promotes the idea of resource optimization.^33^

Several interpretations^34^ have been proposed for the implementation of fog nodes and their configuration, either via servers, networking devices, cloudlets, base stations, or vehicles. With the increasing demand of providing smart solutions, the shift towards utilization of fog gateway devices for edge analytics is being supported by an increasing number of industrial^35^, ^36^, ^37^ IoT platform developers and solution providers, including IBM, Intel, and Microsoft.

The suitability of fog computing in context of IoT has been studied by Sarkar et al.^38^ The approach not only optimizes the use of available in‐network compute and storage resources^39^ but also reduces the bandwidth and storage requirements to upload data on cloud.^40^ Chiang and Zhang^41^ survey main research challenges involved in fog and network context of IoT and how fog can help address them. The benefits of combined and continuous fog to cloud architecture have been presented in the work of Ramirez et al,^42^ and their evaluation shows a significant reduction in service response time and power consumption over the conventional cloud. A very recent and detailed state‐of‐the‐art survey specifically focusing on fog computing with current research challenges being faced have been presented by Mouradian et al.^43^ Hao et al^44^ have presented a software architecture for fog environments.

One of the primary limitations possessed by the previously proposed systems is that they take network connectivity for granted, and those who consider that into account do not support real‐time analytics. They follow the technique to process and analyze previously collected data and perform offline analysis, which is not suitable for real‐time tracking and monitoring of dynamic entities such as dairy cows. The gaps with the existing research are that either it has been developed out of the agricultural context or address the issue of network connectivity, analytics, and control in isolation; this has also been identified as key limitations by Zheleva et al.^45^

Another recent survey by Jukan et al^46^ identifies the lack of interoperability provided by such systems and identify the need of developing an integrated system combining edge, fog, and cloud to provide application and services. The authors here also identify that technology solutions with no consideration of interoperability results in vendor lock‐in, which not only hinders innovation but also results in higher costs for the farmer/user.

While there has been an initiated movement towards data‐driven agriculture in recent times for sustainable and productive growth, no prior work focuses on providing an end‐to‐end IoT solution integrating edge, fog, and cloud intelligence specifically in case of smart dairy farming IoT settings. The closest work is as done by Vasisht et al^47^ primarily towards crop productivity, which has its own set of challenges owing to power, operations, and expenditure that make it unsuitable for a small‐scale farm, especially in adverse weather regions.

We position our work as an answer to the issues mentioned above, thus bridging the gap, and providing an innovative way that integrates edge, fog, and cloud computing to provide a solution specifically in case of smart dairy farming in an IoT setup. The novelty from the proposed model comes from the standpoint of view that it has been specifically designed and developed to address a specific vertical of the IoT ecosystem, ie, dairy farming, and within that to address specifically the problems related to animal welfare, and the microservices‐oriented design makes it multivendor interoperable.

## 3 AGRICULTURE AND ICT: LIMITS, OPPORTUNITIES, AND CHALLENGES

**Figure 1:**
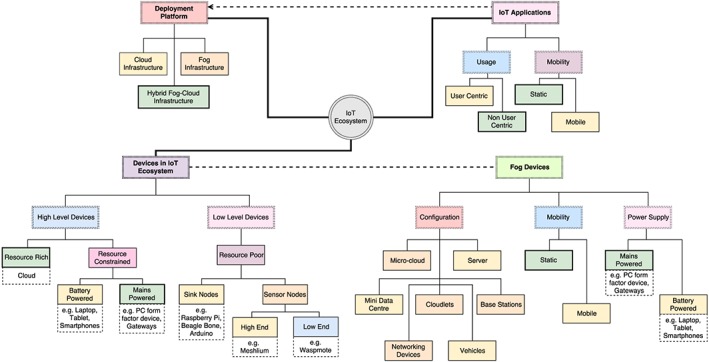
Visual representation of Internet of Things (IoT) ecosystem illustrating IoT devices, available deployment platforms, and type of IoT applications [Colour figure can be viewed at wileyonlinelibrary.com]

Being the key enabler for ICT in smart scenarios, IoT is transforming the way we live and make decisions. Being a multidisciplinary ecosystem, scenarios demanding real‐time processing and decision support have been on the increase. Owing to rapid expansion and a vast expanse of possibilities, an IoT ecosystem is hard to define, complex, and difficult to capture.^48^ To this end, a multidimensional visual representation of the IoT ecosystem illustrating IoT devices, available deployment platforms and type of IoT applications has been illustrated in Figure 1.

The major limitations, challenges, and opportunities in the context of agriculture and ICT are described in the following.

### 3.1 Geographical factors, low Internet connectivity and weather‐based outages

What makes a farm scenario unique is the fact that typical farm locations are geographically remote from the urban context and attributed with sparse to low Internet connectivity. The existing IoT systems and applications that address certain specified objectives in the diverse agricultural domain largely depend on Internet connectivity for proper functioning, as the service and application components are traditionally deployed in a cloud‐centric manner in remote cloud infrastructures.

Owing to their typical geographical locations, a vast majority of farms already have limited cellular^49^ coverage. In that terrain, it is not uncommon to be facing long outages in Internet connectivity due to constraints posed by adverse weather conditions, storms, hurricanes, and other natural disasters. All of these scenarios are apart from general network downtimes, which might be because of another variety of reasons such as network maintenance, broken links, faults, and network/security attacks.

### 3.2 Cloud‐based solutions

The existing solutions suggested by industry and academia primarily include a lot of closed‐form cloud‐based solutions that lack flexibility in the management of data and customization owing to on‐demand services and operations. They often tend to get expensive, and the trade‐off between cost and system utility are not wholly profitable to a small‐scale farmer.

### 3.3 Real‐time analytics

One of the key limitations of the existing solutions is the lack of support for real‐time processing and analytics for latency‐sensitive use cases. For example, while dealing with dairy cattle as in our case, a detailed analysis of patterns from the collected data would enable the development of algorithms, which, once deployed on the fog node closer to the data source, would be able to identify latency‐critical scenarios in real time. The existing systems of historical, cloud‐based analytics are incapable of serving a time‐critical situation, and the bouts of no connectivity further worsen this management.

### 3.4 End‐to‐end solution and vendor lock‐in

Most of the solutions only focus on one tier of the problem, ie, either data sensing, data analytics, or consumer relations.^50^ Architecturally, there is also a major issue of vendor lock in, wherein devices of multiple vendor systems are incompatible with each other, and the lack of features within one solution cannot be complemented with its integration with another.

### 3.5 WiFi sensors: cost and operational trade‐off

Not only is reading data and supporting sensors with WiFi capability challenging in such a remote scenario but also comes at a higher trade‐off with the cost. Supporting sensors with an Internet connection hikes their price for both the acquisition and as well as in terms of operational expenses, while Internet connectivity in itself is a challenge in remote regions of operation.

A farm environment thus poses an atypical use case with heterogeneous demands, and the lack of a flexible solution impacts the acceptance of ICT towards what can otherwise be a very fruitful and profitable venture for both the farmer and the solution provider.

### 3.6 Existing solutions and challenges

There have been several propositions towards enabling connectivity on a farm scenario, as listed in the following.

#### 3.6.1 Microwave wireless terrestrial link

Being a point‐to‐point connection, this technology is used by Internet service providers (ISPs) and cellular carriers to connect remote regions towards a backhaul network for last mile connectivity. This link can connect an ISP to a farm, which can then be distributed locally via WiFi access points. However, this has its own sets of challenges and dependencies and is more dependent on Internet providers rather than the individual farmer. Terrain and physical obstacles also impact such a connectivity, as does rain fade and other weather‐based factors.

#### 3.6.2 TV white spaces

To enable connectivity on the farm, solutions such as FarmBeats^47^ have proposed the use of unlicensed TV white spaces to set up a link from the farmer's home Internet connection to an IoT base station on the farm, which further provides a WiFi front end. However, this involves its own set of challenges and expenditures that might make such a solution difficult for adoption by a small‐scale farmer. For instance, the IoT base station needs power to work, which in itself is a problem. One can use solar electricity, but that is highly reliant on weather conditions and not reliable at this stage, considering the fact that no power on the IoT base station means no WiFi connectivity, and in turn, no data collection and transmission from sensors deployed.

This significantly reduces the reliance and adaptability of ICT on a farm and is not dependable in case of an adverse scenario. Also, currently being unlicensed, it requires permission and authorization from government and federal agencies, which is an additional overhead, thus making it difficult to be used in a number of countries at the moment.

#### 3.6.3 Data mules

A data mule is one such proposition that addresses the issue above in certain use cases where moving objects such as vehicles or people serve as a data mule to collect data from the sensors and to upload it to the cloud as soon as Internet connection becomes available. It was initially proposed for the purpose of delivering emails to remote, rural, or economically underserved regions,^17^ which suffer from the Internet connectivity challenge as specified above. The idea is not straightforward applicable and feasible with large‐scale sensor deployments, as in such scenarios, the storage capacity of microcomputers or on‐chip computers available on sensor devices might get overwhelmed and overutilized with that amount of data. Furthermore, even when the Internet connection is available, the bandwidth available could still be very low, thereby making the upload of large amount of data (in order of megabytes or gigabytes) coming from wide‐scale deployments of data infeasible.

## 4 SERVICE ORIENTATION IN THE IOT ECOSYSTEM AND MICROSERVICES‐BASED ARCHITECTURE

The IoT ecosystem possesses some unique requirements for the infrastructure to meet, few of which have been listed in the following.

- **Specified objective:** Typically, the data generated by real‐world IoT deployments need to be orchestrated to accomplish a specific objective. IoT services are essentially provided by physical devices present on premises and connected to the Internet.
- **Heterogeneous nature/multivendor interoperability:** The devices in an IoT setting may come from a variety of vendors and thus be widely heterogeneous in terms of characteristics, communication protocol, and networking technologies.
- **Variable requirements:** The latency constraints and data requirements of the varied components of an IoT computing systems might differ extremely and thus demand isolation support for each of the components to fulfill their needs and demand accordingly.

With such diverse and critical requirements, we can clearly infer that a monolithic application design approach would no longer be able to handle the unique requirements imposed by certain IoT use cases and would not be the best fit to handle the data that is produced and consumed by individual “things” and users in the IoT ecosystem. Hence, we believe that a microservices‐based approach would be adept at the development and deployment of IoT applications over fog‐enabled infrastructures. This involves a proposed change in the conventional application design and development, whereby an IoT application can be decomposed into a collection of microservices, which can be distributed across physical resources available in the cloud and on the network edge.

Ever since the introduction and adaptation of DevOps in practice, the microservices architectural style is the first realization of a service‐oriented architecture in this context, and with the plethora of advantages that fall in line with the flexible future applications and service, it is rapidly evolving to be the standard for developing continuously deployed systems.

Some key reasoning outlining similarities in the objectives of microservices and IoT are as follows:

- lightweight communication
- independent deployment facility
- bare‐minimum centralized management and control
- isolation support
- building one or multiple applications from a set of different services
- technological independence and support for multivendor interoperability

**Figure 2:**
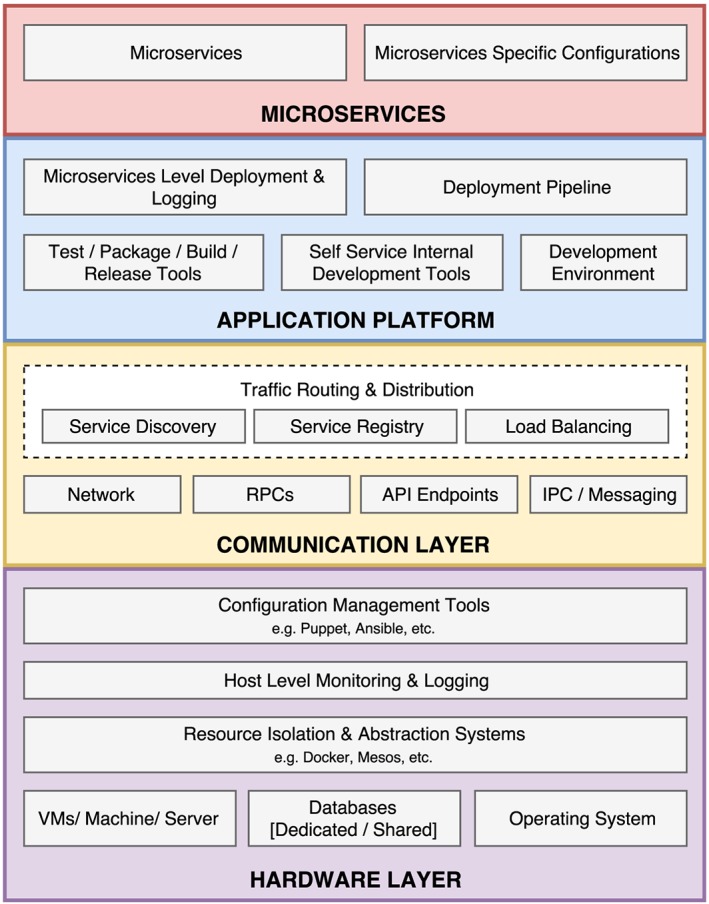
The four‐layer model of microservice architecture. A comprehensive breakdown of what lies in the four‐layered microservices architecture. API, application programming interface; IPC, interprocess communication; RPC, remote procedure call [Colour figure can be viewed at wileyonlinelibrary.com]

The microservices architectural model can be depicted to be an abstraction of four^51^ layers: hardware, communication, application platform, and microservices. Each of these layers comprises of components, as shown in Figure 2. One of the key takeaways of the microservices architecture is technological independence; each service is independent in terms of deployment and platform and, potentially, the technological stack. They can run their own tasks and processes, and communicate via lightweight protocols and application programming interfaces (APIs). This enables each service to be a business capability that can potentially use its own technological stack but still be a part of a connected comprehensive application structure towards a solution to a larger use case.

It is also important to apply analytics over the data to get the required information at the right time and in the right place, depending on the use case. Data in IoT deployments typically flow from things to cloud, with fog acting as middleware. It is critical to apply required data analytics at early stages of the pipeline, and the decomposed microservices of an IoT application can provide visualization support for real‐time local data processing happening on a fog node via API exposure.

Notably, a microservices‐based approach for software development and deployment is currently in wide use by the industry^52^ at production level as a part of best DevOps practices.^53^ However, that approach has been limited to cloud‐based applications only, ie, deploying all the developed microservices for an application within the data center itself, and very limited work^54^, ^55^ has been done that focuses on the deployment of applications across the cloud and network edge, especially in the case of IoT applications. The cloud‐based applicability of a microservices‐style architecture to design a smart city IoT platform has been presented by Krylovskiy et al,^56^ where they share their experience of implementing microservices approach in the cloud environment. However, all such cases so far have only been limited to the cloud and not explored in context of the fog to assist distributed IoT scenarios in real time, or as facilitators to enable dynamic characteristics in the network for service provisioning.

## 5 SMARTHERD MANAGEMENT IOT PLATFORM

In order to address the challenges of remote farm scenarios, we propose the SmartHerd management IoT platform, which is a microservices‐based fog computing–assisted IoT platform towards smart dairy farming.

### 5.1 Goals and objectives

In building SmartHerd, we target the following goals and objectives:

- **Availability:** The downtimes should be minimized, and key tasks such as data collection, processing, and alerts should be resistant to outages.
- **Low dependence on cloud:** Since a primarily cloud‐based application requires a pervasive high‐bandwidth Internet connectivity, the objective is to reduce the amount of data that is sent to the cloud for computation and to construct the services in a way that outages and constraints in connectivity to the cloud does not adversely impact the deployment scenario.
- **Flexibility:** In case of outages, the devised platform should still be able to collect and process data, as well as support alternative methods of alert.
- **Latency‐critical support:** When in a mature stage, the deployment would generate enough periodic data to enable the development of algorithms to detect and identify latency‐critical use cases on the farm. The devised platform should be able to potentially support these latency‐critical use cases that would arise.

### 5.2 Smart dairy farm setup: real‐world deployment

**Figure 3:**
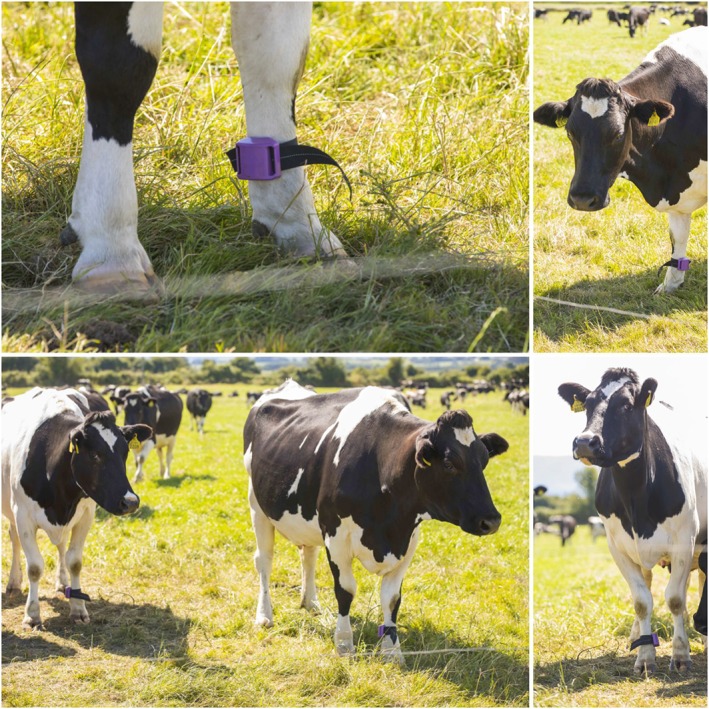
Pedometer attached as a part of the experiment to the front leg of the cows [Colour figure can be viewed at wileyonlinelibrary.com]

The trial 1The ethical approval for the experimentation was taken from Research Ethics Committee of Waterford Institute of Technology, Ireland, prior to the deployment in July 2017. conducted on a local farm with a full dairy herd of 150 cows in Waterford, Ireland. Out of 150 cows used in the trial, only 147 cows were used in the analysis presented in this article. Given the number of options for the sensors/wearables available for livestock monitoring, we chose a radio‐based Long‐Range Pedometer (LRP) (433 MHz, ISM band) over WiFi‐based sensors, considering it does not rely on Internet for its operation, and serves the purpose of data acquisition in farms with low Internet connectivity and frequent outages. Such sensors involve less operational expenditure, and do not use WiFi‐based connectivity to send sensed data to a base station. Therefore, as a part of the deployment, commercially available LRPs (ENGS Systems^© ®^, Israel) specifically designed for use in dairy cattle were attached to the front leg of cows, as shown in Figure 3.

### 5.3 Architecture design and system overview

**Figure 4:**
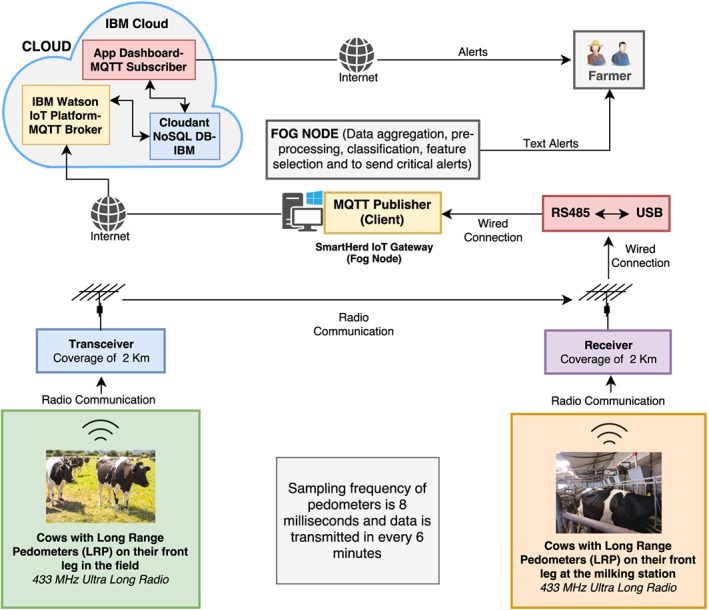
SmartHerd management system overview. DB, database; IoT, Internet of Things; MQTT, message queue telemetry transport [Colour figure can be viewed at wileyonlinelibrary.com]

The overall architecture of the test bed is shown in Figure 4. The pedometer consists of an active system with a (backup) data retention capacity of up to 12 hours that measures the activity of cows (such as standing, lying, walking, etc) with a sampling frequency of 8 ms, and the thereby generated data unit is sent to the corresponding receiver and transceiver in every 6 minutes. The range of the antennas attached to the receiver and transceiver is 2 km each, which gives enough coverage to collect data from cows at all times, whether they are grazing in the field, present in their sheds (during adverse weather conditions), or being milked at the milking station.

As shown in Figure 4, the receiver is the master unit, which sends the received data to the communication unit (RS485 to USB) through wired connection, which in turn then sends it to the SmartHerd IoT gateway (a PC form factor device in our case, which acts as controller and fog node; configuration 2The minimum suggested configuration for the given setup is a dual‐core processor @ 2.3 GHz, 4.07 GB RAM, and 100 GB local storage. used Intel^®^ Core^TM^ 3rd Generation i7‐3540M CPU @ 3.00 GHz, 16.0 GB RAM, and 500 GB local storage) through wired connection via a USB interface.

Most of the dairy farms that are IoT enabled (ie, sensor enabled/smart) have some kind of a farm management system available at hand which the farmer uses to maintain logs or to keep some details. So, our idea was to use the computational resources that are already available in such a setting and leverage them under the fog computing paradigm, so we developed the system (ie, codebase/software framework) as a downloadable piece of software which will run on any PC form factor device. In our case, the laptop available with farmer was with those configurations; hence, we kept the details in the manuscript as it is, and suggested the minimum configuration which will work both in case of PC or as per the industry standard^57^, ^58^ commercially available gateways.

We have chosen Message Queue Telemetry Transport (MQTT)^59^ as the connectivity protocol between fog node (ie, local PC) and cloud (service instances running on IBM Cloud) in our deployment setting. MQTT is an open‐source protocol originally invented and developed by IBM.^60^ It is a lightweight publish‐subscriber model–based protocol designed on top of the transmission control protocol (TCP)/Internet protocol stack.

It is specifically targeted for remote location connectivity with characteristically unreliable network environments such as high delays and low bandwidth,^61^ which is one of the issues in remote farm‐based deployments such as ours. Hence, we chose MQTT as the connectivity protocol in our deployment.

MQTT provides three quality‐of‐service (QoS) levels^62^ that define the guarantee of delivery for a specific message between sender and receiver:

- At most once (QoS 0): There is no guarantee of delivery here. If the failure happens, no additional attempts are made to rehandle those messages. It is often called “fire and forget” and provides the same guarantee as the underlying TCP.
- At least once (QoS 1): At least once means that messages in a stream are guaranteed to be delivered at least one time to the receiver. If the failure happens, additional attempts will be made to rehandle those messages. This approach may cause unnecessary duplication of data packets in the streams.
- Exactly once (QoS 2): Exactly once means that messages are guaranteed to be handled exactly the same as it would be in the failure‐free scenario, even in the event of various failures. It is the safest and slowest QoS level.

A developer can specify the QoS level that they want on both publisher and subscriber as per their application requirements.

The MQTT architecture comprises of two components, namely MQTT clients (such as publishers and subscribers) and MQTT broker (for mediating messages between publishers and subscribers). In our setup, these components are as follows:

- **MQTT publisher:** Script running on fog node (ie, SmartHerd IoT gateway).
- **MQTT broker:** IBM Watson IoT platform (as a service on IBM Cloud).
- **MQTT subscriber:** Application designed and hosted on IBM Cloud.

Thus, the data from the fog node after classification as described above is streamed to IBM Watson IoT platform using MQTT; the IBM Watson IoT platform receives all these messages, and the MQTT subscriber listening to the events of this broker picks up all the data and stores it in Cloudant NoSQL JSON Database (Database service on IBM Cloud).

### 5.4 Microservices‐oriented application design

**Figure 5:**
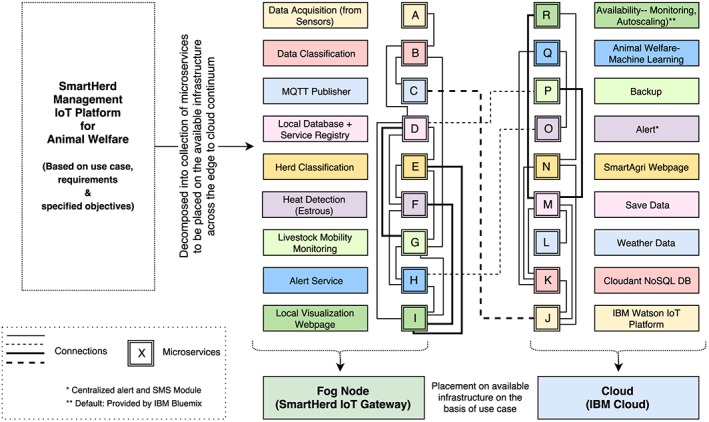
Decomposition of SmartHerd Internet‐of‐Things platform in terms of microservices and placement of these services across edge of the network and on the cloud. DB, database; MQTT, Message Queue Telemetry Transport [Colour figure can be viewed at wileyonlinelibrary.com]

The desired application has been built as a collection of microservices as shown in Figure 5. We envision microservices as one of the key enablers while building an end‐to‐end IoT solution in such scenarios.

We make the key observation that data requirements and latency constraints of various applications and services in a smart dairy farming scenario can be primarily classified in the categories as shown in Table 1. This along with other reasons mentioned previously motivates the use of microservices‐based architecture and fog computing–based approach in smart dairy farm setup.

**Table 1:** Data requirements and latency constraints

**Application/Service**	**Latency Constraints**	**Data Requirement**
Query sensor data	Minutes	Immediate
Livestock location monitoring	Minutes	Immediate
and mobility analysis		
Heat detection	Hours	Immediate
Lameness and other	Hours ‐ Days	Nonimmediate
Illness detection		
Animal health statistics	Hours ‐ Days	Nonimmediate
Logging and other application	Hours ‐ Days	Nonimmediate
Performance logging services		
Animal behavior and variability	Days	Nonimmediate
Analysis via mathematical modeling		

From an application deployment standpoint, fog computing can be perceived as component of application running at the network edge as well as in cloud, with the components running at fog being latency sensitive and time critical in nature. As fog computing was initially aimed to improve network latency^63^ between sensors, applications and users; however, coupled with a microservices‐based application design, we also envision it as an enabler to provide services that efficiently utilize the fog‐enabled infrastructures, while also catering to situations where Internet connectivity is constrained.

For ease of orchestration, fog services are abstracted inside containers. The realization of service abstraction becomes more important in scenarios where fog devices are heterogeneous in nature and can range from end‐user devices to access points, to routers, and to switches; so to accommodate such heterogeneity, service abstraction is desired and can be realized in terms of containerization. The examples of container technologies include Java Virtual Machines (JVM), Python Virtual Machine (PVM), Linux containers, and Docker 3
https://www.docker.com/
. A wise decision on deciding which container technology to choose acts as an important factor in the efficiency and performance of the overall system. In our deployment setting, we used PVM and JVM as container technologies (or as equivalent possible substitute) on fog node and the default available container technology in the IBM Cloud (ie, Docker as platform and Kubernetes as container orchestration system for Docker containers) while building SmartHerd IoT system.

We wanted to keep the service management overhead on a fog node as low as possible. The programming platform (languages) used in the development process were Python and Java. Keeping track of developed modular part of the code base as isolated processes (Namespaces and Cgroups) gave us a way to use them as individual and different services with a simple service registry (in form of a simple file) rather than using an additional software/framework to do the same; hence, we used PVM and JVM as containers equivalent on a fog node.

The designed and built microservices of SmartHerd IoT ecosystem are placed across network edge at SmartHerd IoT Gateway and Cloud, ie, IBM Cloud in a manner that latency‐sensitive one and the ones with immediate data requirement are placed at the fog node and the remaining ones are placed at the cloud with the desired resource requirement^40^ of each of the service being met in an efficient manner.

### 5.5 Workflow

**Figure 6:**
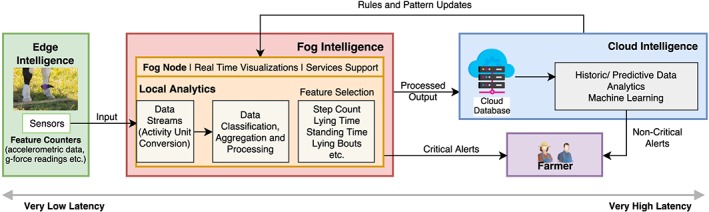
Workflow and data flow in the test bed deployment [Colour figure can be viewed at wileyonlinelibrary.com]

The detailed work flow of the experimental setup is as shown in Figure 6. The fog node also provides a dashboard for the farmer and serves as a visual medium to see the event information and other related sensor data. After gathering initial insights from the collected data and generating the corresponding alert/response, the aggregated, processed data are sent to the cloud for historical storage and analysis. The cloud is also the site for fusion of the data from other sources, such as weather data. The learning pattern from historical data analysis at cloud is sent back to the fog node for further enhancement of the system and to increase the overall efficiency and responsiveness.

Since fog devices have limited computational capabilities, so in such circumstances, it makes sense to have module‐based data analytics components that can be remotely pushed onto fog devices dynamically and on demand. All the long‐term information and data are stored in the cloud, and like the fog, it too provides a dashboard where the farmer can input and modify any relevant information related to their livestock and demand further features and services.

### 5.6 Use case

As shown in Table 1, while the data categorized under nonimmediate requirements is given low priority for fog‐level computation and might even be provisioned to the cloud, the main point of focus is the immediate attention and actionable decisions on the data that have immediate requirements of processing for a critical output towards the welfare of the animal.

To determine the attributes of Table 1, we conducted a survey of dairy farmers and dairy‐tech service provider companies and consulted the literature. The more insight on this came from survey than from literature. This is in line with the domain‐driven design methodology of software development, ie, to work closely with a domain expert (majorly the farmer in our case) and help us understand better how the real‐world system works and what are the demands/needs there.

In our use‐case scenario, the latency constraint and data requirement of a microservice remains static all the time. However, this might not be the case with other use case scenarios; as a hypothetical example, here, consider a microservice that takes temperature and humidity values from the sensors to determine environmental conditions, for example, in order to regulate air conditioning in an area. Now, a sudden change in temperature might not mean anything, but if the change stays for a long time, it might be an indication of fire in that area. Now in this scenario, the latency constraint and data requirement of the microservice will change. So, these two features of a microservice are use‐case dependent.

**Figure 7:**
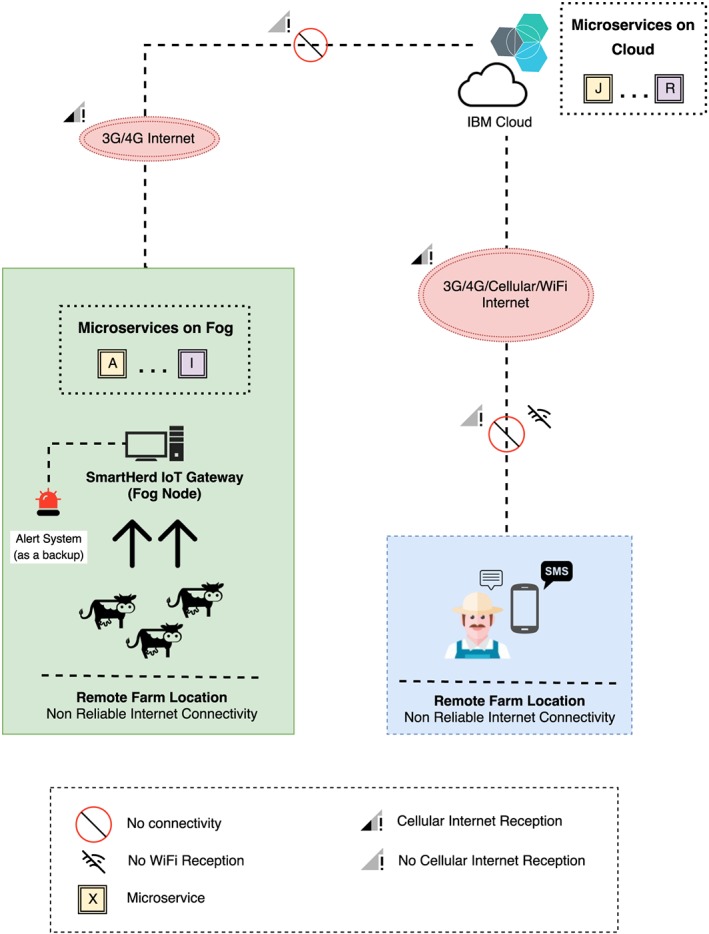
Use‐case scenario with constrained Internet connectivity [Colour figure can be viewed at wileyonlinelibrary.com]

In the case of detection of an event that would require attention towards the animal, an alert is sent out to the farmer. In summary, SmartHerd, a dairy farm use‐case solution towards a smart farm deployment, is as shown in Figure 7.

## 6 RESULTS AND ANALYSIS

A brief demo video of the developed system is available at this web‐link.^64^

### 6.1 Herd classification and cow profiling

For the system to differentiate between normal and anomalous behavior, there is a need of cow profiling. There needs to be a baseline for the normal activity in the herd, which characterizes normal behavior. Animals grazing within the same pasture can influence the movement, grazing locations, and activities of other animals randomly, with attraction, or with avoidance^65^, ^66^, ^67^; therefore, most of the animals will have their activity levels almost equivalent to the herd mean. For such reasons, any deviation from such behavior is classified and analyzed for an anomaly. Such an analysis eliminates the effects of external factors as these will be largely affecting the herd as a whole and only leaves back exclusively the individual behavior of the cow.

As a part of our monitoring and analysis, we use the data of three activities for each cow:

1. **Step count:** The number of steps an animal makes per day. This depends on the age, the level of activity of the cow, and familiarity with the pasture.
2. **Lying time:** The number of hours an animal spends lying down. Lying time has a crucial role to play in determining the emotional and physical health status of a cow, and is cumulative of rumination, sleep, rest, and other factors, which include state of pregnancy, medical conditions, range of illnesses, and potential lameness.
3. **Swaps per hour:** The number of times an animal moves from lying down to standing up, per hour.

In the following sections, we describe the underlying technique used to form cow profiles to detect anomalies in the herd.

### 6.2 Age‐based classification

**Figure 8:**
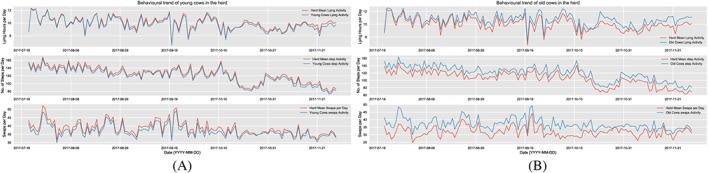
Young and old cows. A, Behavioral trend of young cows for the three activities in the herd; B, Behavioral trend of old cows for the three activities in the herd [Colour figure can be viewed at wileyonlinelibrary.com]

As mentioned earlier, 147 dairy cows were used in this analysis from the herd consisting of 150 dairy cows in our experimental setup, the age distribution of which are as depicted in Table 2. The first and most intuitive way of clustering the cows is in terms of age groups: young (age 2 to 5) and old (age 6 to 12). While this matches the intuition that the activity patterns of young cows are closer to the herd mean as opposed to older ones in the herd, there are crucial aspects missed out. For instance, the sample size in young category exceeds the old by 67%, and it is difficult to trace out the subsets of animals that may behave differently in these binary groups. It thus excludes the outliers in each group and generalizes the classification as a whole, which leaves the true behavior of a cow difficult to trace. The behavioral trend of young and old cows for the three activities is as shown in Figure 8A and Figure 8B, respectively. Our initial work has presented age‐based clustering of cows combined with data analytics to detect anomalies in their behavior and microservices‐based application flow for integration of specific services such as lameness in dairy cattle.^68^, ^69^

**Table 2:** Age distribution of cows in the herd

**Age (in years)**	**Number of Cows**	**Category**
2	29	
3	31	Young cows (age 2 to 5)
4	29	Number of young cows = 111
5	22	
6	9	
7	7	
8	7	Old cows (age 6 to 12)
9	5	Number of old cows = 36
10	7	
12	1	
**Total**	147	147

### 6.3 Activity‐based clustering

Although behavioral studies indicate that cattle in the same pasture are not considered independent experimental units because of the potential confounding effects of the herd's social interactions,^66^ the same authors concluded that under some situations such as larger herds (of around 53‐240 cows), movement patterns of subsets of individual cows may have a level of independence that is sufficient for analysis as individual experimental units.

Therefore, there can be no one‐size‐fits‐all solution for determining the behavior of an individual cow, considering the difference in the behavior of subsets within the same herd. Not all cows would have the same levels of activity as others their age or behave similarly.

For the purpose of a better analysis of activity, we calculate the absolute deviation (AD) for each of the three monitored activities for each cow against the herd mean, as illustrated in Equation 1.

(1) $$C_{\text{AD}}=|H_{m}-C_{a}|$$

**Figure 9:**
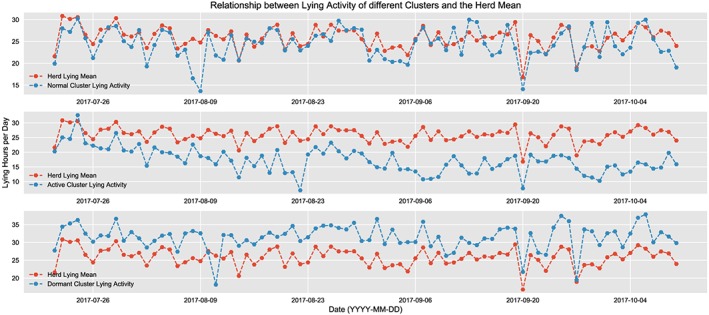
Lying activity of the clusters against herd mean [Colour figure can be viewed at wileyonlinelibrary.com]

**Figure 10:**
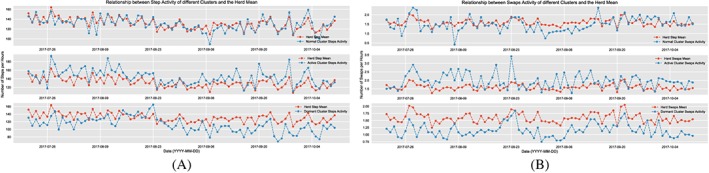
Step and swap activity of clusters. A, Step activity of the clusters against herd mean; B, Swap activity of the clusters against herd mean [Colour figure can be viewed at wileyonlinelibrary.com]

Here, *H*_*m*_ is the herd mean on a given day, *C*_*a*_ is the cow's corresponding activity, and *C*_AD_ is the AD of cow. Based on a best of three analysis of these ADs for each of the activities, we classify the herd into three clusters based on their behavior: active, normal, and dormant. The cows in the active cluster have an activity level higher than the herd mean, ie, typically higher values of step count and smaller values of lying time. The cows in the normal cluster have their activity levels almost equivalent to the herd mean, and finally, those in the dormant cluster have lower values of step count and spend most of their time lying down. Figure 9 shows the lying activity of the different clusters against the herd mean, and Figure 10A and 10B depict the same for number of steps and swap count, respectively.

This method of classification is unbiased of age; thus, it not only covers the outliers within each age group but also depicts a more in‐depth analysis of the activities of each of the cows belonging to each age group, and the overall analysis of the herd activity. The distribution of each cluster against the age is as shown in Table 3. The key observations here include the following:

- The active cluster comprises of 30% young (2‐5) and 7% old (6‐12) animals in the herd.
- The normal cluster comprises of 45% young (2‐5) and 12% old (6‐12) animals in the herd.
- The dormant cluster comprises of 0% young (2‐5) and 6% old (6‐12) animals in the herd.

**Table 3:** Distribution of each cluster against the age

**Age (in years)**	**Active**	**Normal**	**Dormant**
**2**	12	17	0
**3**	9	22	0
**4**	14	14	1
**5**	9	13	0
**6**	7	2	0
**7**	2	4	1
**8**	0	4	3
**9**	1	1	3
**10**	0	6	1
**12**	0	0	1
**Total**	54	83	10

### 6.4 Animal welfare alert system

**Figure 11:**
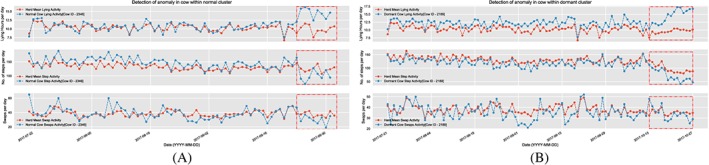
Detection of anomalies in clusters. A, Normal; B, Dormant [Colour figure can be viewed at wileyonlinelibrary.com]

Postmonitoring, analyzing, and clustering the cows on the basis of their activity, the SmartHerd platform addresses the critical use cases in a dairy farming scenario where the farmer is alerted about a cow's activity that requires attention and intervention. As shown in Figure 7, the system uses a short messaging system–based service to alert the farmer in this context and, in the case of low cellular/Internet connectivity, displays a physical audio‐visual alert on the farm premises. The alerts generated include the following:

- **Heat detection (Oestrus):** One of the most profitable and important activities for a dairy farmer is herd reproduction. This is not only important to sustain milk production throughout the years but also to increase the size of the herd. A cow's milk yield gradually decreases after the initial months postcalving, hitting a dry state eventually. Considering such scenarios on a dairy farm, it is critical to accurately identify an animal in heat, which is the best time for insemination of a cow towards pregnancy. This is a 21‐day cycle in cows, and the exact heat window only lasts once for about 18 hours in this 3‐week cycle, which makes it critical to be identified in time. One of the more accurate methods of detecting an animal in heat is monitoring the step activity, which is an increasingly restless behavior with a sudden spike in step activity, characterizing the beginning of the heat (oestrus) window for the cow, for which the farmer needs to be alerted in time for action to be taken before the insemination window ends.
- **Monitoring of an animal's migration between clusters:** Not all cases of a cow being dormant mean that there could be a cause of concern—there might be cows that are dormant and perfectly healthy, as long as they are consistent in their clusters' behavior over time. While the cows are liable to gradually shift their behavior over time between clusters, what is a potential cause of concern is the sudden movement of an animal from a high or normal activity level to being dormant or a sudden drop in its activity even while being dormant. This could indicate a sudden illness, infection, or more commonly, lameness. For example, Figure 11A shows a rapid movement of a cow from being normal to dormant, and Figure 11B shows a sudden drop in the activity of a dormant cow. The farmer was alerted on these cows' behavior, and upon inspection, they were found to be developing lameness.
- **Calving alert:** When nearing the time of birth, a pregnant cow tends to find a secluded spot away from the herd and rest, showing an increased lying time. Therefore, lying activity, when tracked with insemination date and pregnancy stage, can quite accurately alert the farmer towards calving activity for a cow.

### 6.5 System resilience

#### 6.5.1 Network outages

Since the SmartHerd IoT platform does not depend on WiFi sensors for data collection, the data collection is not impacted due to Internet constraints, outages, or connectivity issues. Further, the fog node is responsible for the local processing of the data input from sensors and the identification of critical activities such as heat detection. This ensures that critical alerts are not missed out in the state of constrained connectivity to the cloud, and the platform is still able to monitor and analyze the behavior of the herd and track animals' welfare.

**Figure 12:**
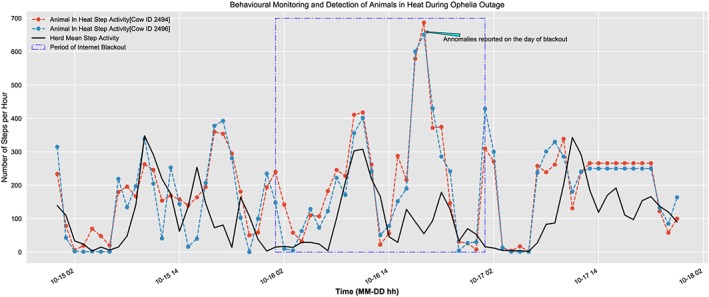
Working of system during hurricane Ophelia outage [Colour figure can be viewed at wileyonlinelibrary.com]

A true test of this platform came up at the time Ireland was hit by Hurricane Ophelia^31^, ^32^ on October 16, 2017 and the farm location in county Waterford lay in the most severe red alert zone. The area was impacted widespread outages of both power and Internet for two days, and as shown in Figure 12, during the time of the blackout, the platform still remained operational, collected and analyzed the data, and was able to capture the animals in heat at the time and alert the farmer. Thus, even though it was unable to sync up with the cloud due to no connectivity, the SmartHerd IoT gateway was able to function uninterruptedly for a reasonable period of time and provide functionalities even when the connection to cloud was lost, and functioned towards the welfare of the herd.

#### 6.5.2 Failure/overload situation of fog node

In the current setup, we only considered one gateway. In the likely event that the gateway is overloaded or is unavailable, the sensors have a data retention capacity of up to 12 hours, the gateway has a local database that stores the data before preprocessing, the MQTT publisher saves the state of the last successfully transmitted events, and would then continue from where it stopped in case of a network outage. Another possibility to increase resilience of the system is to have coexistence of multiple gateways in the infrastructure, which are synchronized with each other, and data can be redirected between them in case of failure of one gateway; this is something we are considering in our future work.

### 6.6 Data reduction

**Figure 13:**
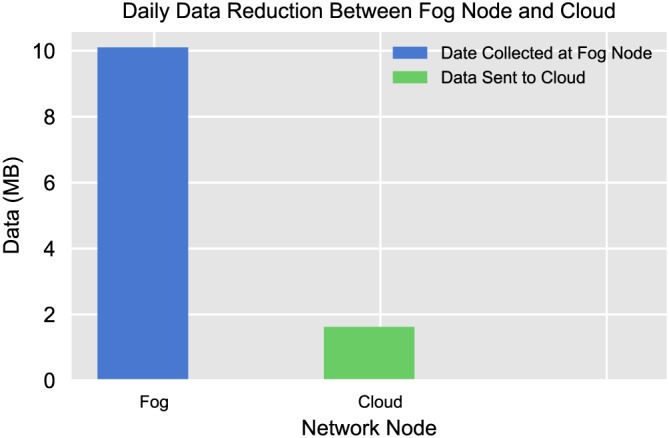
Daily reduction in the amount of data between the fog node and the cloud [Colour figure can be viewed at wileyonlinelibrary.com]

From what we have monitored, each sensor/LRP generates around 70 KB of data per day, which, as shown in Figure 13, accounts for 10.1 MB of data collected from the 147 sensors at the SmartHerd gateway each day, with data acquisition and streaming happening every hour. However, given the local processing capability attributed to fog computing, the amount of data that is sent further to the cloud is only 1.62 MB per day, which, as shown in Figure 13, is an 84% reduction in the amount of data that would otherwise have streamed to the cloud throughout the day. This aspect of data reduction becomes even more crucial while scaling up the farm and the herd, as the amount of data collected and streamed would rapidly increase.

### 6.7 Accuracy of the system developed

We formulated our problem as a binary‐class classification problem with irregular (anomalous behavior) being the positive class and regular as the negative class. The data split was made as 80%‐20%, ie, 80% of data was used for model training and the remaining 20% was used for testing.

We experimented on a number of classification algorithms ranging from support vector machine, random forest (RF), K‐nearest neighbors, and decision trees while noting the accuracy. The best two performing were RF and K‐NN with an accuracy of 91% and 1 day before visual signs and 87% with 3 days before visual signs, respectively, as presented in Table 4.

**Table 4:** Accuracy of the developed system

**Classification Model**	**Accuracy**	**Number of Days**
	**(in %)**	**Before the Visual Signs of Anomaly Appears**
Random forest	91	1
K‐Nearest NeighborsŠ	87	3

Optimal results for K‐NN were obtained at *k* = 4, which gave an accuracy of 87% with 3 days before the visual signs could be seen. In all, the normal cluster model had a sensitivity of 89.7% and specificity of 72.5%. The anomalies reported by the system were confirmed as lame cows by the farmer.

### 6.8 Resource utilization at fog node and discussion on platform performance on using fog node with low‐level computational power

The main processing component among the microservices running on the fog node is the MQTT publisher, which is responsible for data preprocessing, aggregation, and streaming processed data to the cloud. We monitored resource (CPU and memory) consumption on the fog node in a time window of every 3 minutes when all the microservices were running on it. Using the psutil (process and system utilities)^70^ Python library, we calculated the percentage increase in both CPU and memory before, during, and after streaming (ie, before the MQTT publisher was run, during, and after it was run). We used the percentage increase because this would form a baseline here, ie, irrespective of change in the fog node capabilities, the same increase in resource consumption would be expected.

**Figure 14:**
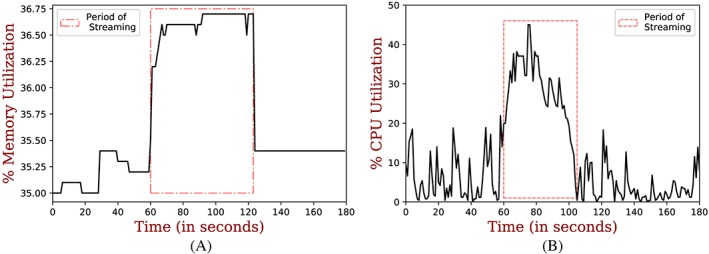
Resource utilization at fog node (ie, SmartHerd IoT Gateway). A, Memory utilization at fog node; B, CPU utilization at fog node [Colour figure can be viewed at wileyonlinelibrary.com]

Figure 14A shows memory usage before, during, and after the MQTT publisher is run. The region highlighted shows the period during which the MQTT publisher was streaming. As it can be seen, there is an increase from about 35.38% to 36.75%. The total usable memory was about 14.50 GB. This would mean the impact of MQTT Component was only 0.12 GB.

Figure 14B shows the CPU usage before, during, and after the MQTT publisher is run. The region highlighted shows the period during which the MQTT publisher was streaming. As it can be seen, there is an increase from about 20% to 45% in CPU utilization.

Table 5 summarizes the CPU and memory utilization before, during, and after streaming.

**Table 5:** CPU and Memory utilization on fog node before, during, and after streaming for comparison of the developed system to be able to run in resource‐constrained environment as well

**Resources**	**Before Streaming With All**	**During Streaming With All**	**After Streaming With All**
	the Microservices Running	the Microservices Running	the Microservices Running
**CPU**	At max 20% and	From 20% to 45 %	At max 20% and
	mostly below that	(25% increase)	mostly below that
**Memory**	At max 35.38% and	From 35.38% to 36.75%	At max 35.38% and
	mostly below that	(1.37% increase)	mostly below that

In all, the overall resource utilization and effects of MQTT component on the fog node are very minimal for it to run on very resource‐constrained compute environments.

Although the real‐world experiment was carried on a high‐end fog node (computer), but prior to the experiment (ie, before putting the developed code base in the actual deployment), we tested resource (CPU and memory) consumption on another PC setting (Intel^®^ Core^TM^ i5‐4300M CPU@ 2.60 GHz, 8.0 GB RAM, and 500 GB Local Storage), and also on a single virtual machine instance running on OpenStack with following configuration: two VCPUs (Intel^®^ Xeon Processors @2.60 GHz), 4 GB RAM, 20 GB disk, initially with artificial data and later with data obtained from ENGS systems (company who provided sensors in the deployment).

A similar behavior as mentioned above was observed on them and the developed system worked well with same settings as in real deployment.

Further, in context of efficient in‐network resource utilization and increasing system resilience, one of the possible directions of future work is to look into distributed learning–based^71^ and distributed data analytics–based 72, 73 approaches in such real‐world IoT‐based deployments.

### 6.9 Service delivery time

The service delivery time here represents the average query time that we performed on the system with different queries, like a farmer can query the system to see the report about a particular animal or to see the system in general. This time also corresponds to the time when an alert is generated or an anomaly is detected, and the farmer needs to be informed then how much time does the developed system take to do that. It includes the network round trip time along with the processing delays by the system. The network available at user's mobile device can change from 2G, 3G, to 4G. Table 6 summarizes the service delivery time of the developed system.

**Table 6:** Service delivery time of the developed system

**Average Query Response Time**	**Network Type Available**	**Network**	**Total Service Delivery Time**
**Total Service Delivery Time**	**on User's Device**	**Round Trip Time**	**(in ms)**
**SmartHerd System**		**(in ms)**	**(Sum of Query Response Time**
**(in ms)**			**and Network Round Trip Time)**
	2G	250‐300 ms	270‐330 ms
20‐30 ms	3G	80‐100 ms	100‐130 ms
	4G	30‐50 ms	50‐80 ms

### 6.10 Power consumption

**Figure 15:**
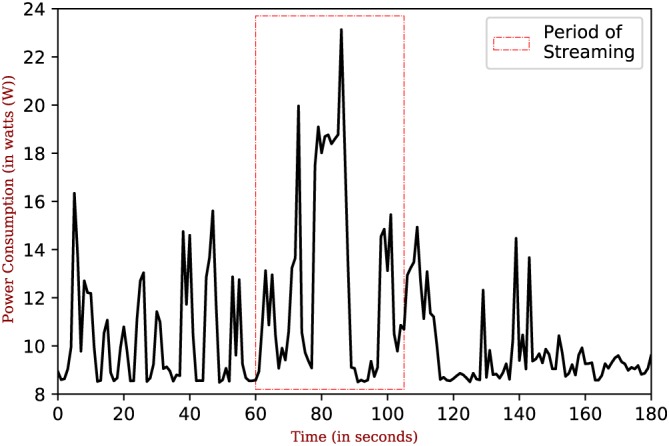
Power utilization at fog node [Colour figure can be viewed at wileyonlinelibrary.com]

We used Intel^®^ Power Gadget tool^74^ to measure the power consumption of the fog node (ie, SmartHerd IoT Gateway). It is a software‐based power usage monitoring tool enabled for Intel^®^ Core^TM^ processors. While we collected the data for a longer period during the deployment for this analysis, we observed a constant power consumption pattern throughout. Thus, to give a better representation of the power consumption pattern, we present the power consumption at the fog node in a time window of 3 minutes in Figure 15. As visible from the plot, the maximum utilization is 23.2 W and for most of the time, it stays below 18 W. The utilization reaches its maximum value (during the streaming period) when there is a message stream happening between the fog node and the cloud node.

### 6.11 Deployment cost

The total deployment cost of the system was $11 711. Table 7 details cost of different components in the deployment.

**Table 7:** Deployment cost

	**Part Description**	**Quantity**	Unit Price (in **$**)	Extended Price (in **$**)
1	WALL TRANSFORMER	3	16	48
	240 Vac IN 24 Vac OUT 500 mA			
2	COMMUNICATION CARD ‐ USB	1	228	228
3	RECEIVER LRP ‐ ENGS KIT	1	760	760
4	ANTENNA OMNI	1	91	91
	433 MHz N‐TYPE 5.5 DBI			
5	ANTENNA OMNI LONG RANGE	1	148	148
	430‐450 MHz 2.3 m			
6	Metal Bracket for	2	24	48
	YAGI/OMNI Antenna			
7	CABEL RF RG213	2	68	136
	N‐TYPEM TO BNCM 8 m			
8	TRACK A COW ‐	150	51	7650
	COWTAG Active LRP V2 (Sensors			
9	2.12.0.3(1.0.6.2)	1	684	684
	ECO HERD SOFTWARE			
10	TRANSCEIVER LRP KIT	1	1280	1280
11	COMMUNICATION	30	2.6	78
	CABLE 485			
12	LIGHTNING	2	63	126
	PROTECTOR + CABLE			
13	SOLAR PANEL KIT	1	224	224
14	Shipment Cost	1	210	210
**Total Cost**				**$** **11 711**

## 7 REAL‐WORLD DEPLOYMENT: LEARNINGS AND PRACTICAL EXPERIENCES

The primary challenge is to design an IoT solution to meet the specified objective given the highly variable, harsh, and resource‐constrained environment in a smart dairy farming setting. This includes making the system resilient and fault tolerant to cope up with the variable farm environments, including weather‐based network outages and connectivity issues because of remote location of the farm. The use of fog computing brings efficiency and sustainability to the overall IoT solution being proposed.

If we are to really envision the future of smart farming, there needs to be a solution that addresses all these challenges that a farm environment can impose. Unlike physical “things” in other IoT use cases, an animal farm environment involves higher stakes due to a direct involvement with cattle and livestock welfare. If we are to increase dependence on technology to further increase the productivity while scaling up, the platform solution needs to be flexible, adaptive, and fault tolerant, keeping the constraints in check.

With respect to our real‐world deployment, deciding which sensors to choose to build the sensing infrastructure, the right application protocols, and using the best software development practices, the overall solution demands a dedicated brainstorming for a reasonable period of time to achieve efficiency and sustainability. The trade‐off between initial costs for the setup of such a system would be balanced in the long term not just with payback via advantages, but also potential savings, including reduced cost of connectivity to the cloud, low‐bandwidth requirements, extended battery life cycles on sensors, etc.

## 8 CONCLUSION

In this work, we propose the emerging fog computing infrastructure as a key enabler for IoT applications and services in situational scenarios of constrained Internet connectivity. We envision a microservices‐based approach to assist applications in such a hybrid fog‐cloud environment, and propose a solution that addresses the connectivity and animal welfare in a smart dairy farming scenario.

In the SmartHerd IoT platform, the use of radio‐based communication instead of WiFi‐based sensors ensures pervasive data collection even in the scenarios of Internet and weather‐based outages, while providing the advantages of decreased cost, longer battery life cycles, and wider coverage of the distribution of animals on the farm. Further, the use of SmartHerd IoT gateway leverages fog computing and enables the intelligent processing of data closer to the source, thereby supporting a real‐time processing and monitoring of livestock. Insights from our real‐world deployment suggest that an activity‐based clustering of animals in the herd allows to monitor the behavior of dairy cows and track their welfare, and alert the farmer towards detected anomalies in the behavior of individual cows, even with cloud out of the active loop.

The system in its overall architecture and design is versatile and better adapted to Internet constraints, outages, and connectivity issues on the farm, thus making it a dependable solution for effective livestock monitoring and management in a smart dairy farming scenario.
